# Molecular Mechanism and Pathways of Normal Human Parturition in Different Gestational Tissues: A Systematic Review of Transcriptome Studies

**DOI:** 10.3389/fphys.2021.730030

**Published:** 2021-09-10

**Authors:** Wenjing Ding, Stephen Siu Chung Chim, Chi Chiu Wang, Caitlyn So Ling Lau, Tak Yeung Leung

**Affiliations:** Department of Obstetrics and Gynaecology, Faculty of Medicine, The Chinese University of Hong Kong, Hong Kong, China

**Keywords:** transcriptomics, parturition, molecular regulation, immune response, inflammatory response

## Abstract

**Objective:** Genome-wide transcriptomic studies on gestational tissues in labor provide molecular insights in mechanism of normal parturition. This systematic review aimed to summarize the important genes in various gestational tissues around labor onset, and to dissect the underlying molecular regulations and pathways that trigger the labor in term pregnancies.

**Data sources:** PubMed and Web of Science were searched from inception to January 2021.

**Study Eligibility Criteria:** Untargeted genome-wide transcriptomic studies comparing the gene expression of various gestational tissues in normal term pregnant women with and without labor were included.

**Methods:** Every differentially expressed gene was retrieved. Consistently expressed genes with same direction in different studies were identified, then gene ontology and KEGG analysis were conducted to understand molecular pathways and functions. Gene-gene association analysis was performed to determine the key regulatory gene(s) in labor onset.

**Results:** A total of 15 studies, including 266 subjects, were included. 136, 26, 15, 7, and 3 genes were significantly changed during labor in the myometrium (seven studies, *n* = 108), uterine cervix (four studies, *n* = 64), decidua (two studies, *n* = 42), amnion (two studies, *n* = 44) and placenta (two studies, *n* = 41), respectively. These genes were overrepresented in annotation terms related to inflammatory and immune responses. TNF and NOD-like receptor signaling pathways were overrepresented in all mentioned tissues, except the placenta. *IL6* was the only gene included in both pathways, the most common reported gene in all included studies, and also the gene in the central hub of molecular regulatory network.

**Conclusions:** This systematic review identified that genes involved in immunological and inflammatory regulations are expressed in specific gestational tissues in labor. We put forward the hypothesis that IL6 might be the key gene triggering specific mechanism in different gestational tissues, eventually leading to labor onset through inducing uterine contraction, wakening fetal membranes and stimulating cervical ripening.

**Systematic Review Registration:** Identifier [CRD42020187975].

## Introduction

Timely and safe parturition is one of the essential elements of a successful pregnancy, which is a key factor in the human reproductive cycle. The initiation of parturition has been characterized by uterine changes including cervical softening and ripening, activation of amnion and decidual membranes, and conversion of uterine smooth muscle from being quiescent to contractile (Smith, 2007). Abnormal timing of triggering these changes leads to adverse pregnancy outcomes. For instance, premature activation causes preterm birth that is one of major causes of neonatal mortality and morbidity (Vogel et al., 2018). Delayed activation may lead to post-term pregnancy, associated with fetal compromise and in-utero death (Cunningham et al., 2014). Excessive stimulation might also cause precipitate labor, resulting in maternal and neonatal morbidity (Cunningham et al., 2014). However, the mechanisms triggering and sustaining human parturition are not fully understood, as this process entails a complex and multifactorial set of events that derive from both the maternal and fetal unit.

Human parturition has been demonstrated as a hormonal event. Placental production of corticotropin-releasing hormone, which is linked to fetal and maternal cortisol positive feedback mechanism, may initiate an endocrine cascade, leading to an exponential increase near term (Frim et al., 1988; Majzoub and Karalis, 1999). Corticotropin-releasing hormone is capable of increasing the actions of oxytocin and prostaglandins, that is inducing uterine contractility (Ravanos et al., 2015). Progesterone is another essential hormone keeping the uterus in a quiescent state during gestation. Although in some animals a decrease of maternal serum progesterone levels is the initiation step of parturition, it is not the case in humans (Zakar and Mesiano, 2011). “Functional progesterone withdrawal” has been proposed by investigators as a potential mechanism in human labor (Brown et al., 2004; Mesiano et al., 2011).

So far, mRNA profile of gestational tissues from samples taken before and after the onset of labor using genome-wide analysis brings molecular insights into the underlying mechanism of parturition. In order to dissect the underlying molecular regulation and pathways of the pregnancy clock and alarms to initiate the parturition, we conducted this review to systematically review and summarize the consistently differentially expressed genes in various gestational tissues of healthy pregnancies before and after labor onset in more than one transcriptomic study.

## Methods

Untargeted genome-wide transcriptomic studies comparing the gene expression of various gestational tissues in normal term pregnant women with and without labor were included. This systematic review was performed and reported according to the guidelines set by the Preferred Reporting Items for Systematic Reviews and Meta-Analyses (PRISMA) statement.

### Search Strategy

We searched Pubmed and Web of Science for articles published from inception to January 2021, using the following combination of terms: (“parturition” OR “delivery” OR “labor”) AND (“gene expression profiling” OR “transcriptome” OR “whole exome sequencing” OR “microarray analysis” OR “oligonucleotide array sequence analysis” OR “gene expression”). Search results were filtered by “published in English.” We also screened the reference citations for relevant studies to identify possible missing publications.

### Study Selection

Publications were included if they were (1) human studies; (2) normal term pregnancy; (3) comparisons between two groups of term pregnant women with and without labor, or comparisons between before and after labor onset in the same group of women; (4) untargeted genome-wide analysis; (5) studying differential gene expression; (6) original article. Studies were excluded if they met any of the following conditions: (1) samples were extracted from women with complicated pregnancy; (2) samples were prepared from cells rather than tissues; (3) tissue samples were treated before RNA extraction; (4) only conference contents or the abstracts were published, or the specific data were missing; and (5) subjects who need to receive medication to induce labor were included.

### Data Extraction

The literature search and data extraction from all eligible studies was independently performed by two reviewers. Any discrepancy in the search result was resolved by discussion between a third reviewer and all participating authors. The following data were extracted if available: (1) study characteristics (authors, publication year, number of subjects, gestational weeks at delivery, mode of delivery, and types, locations and methods of sample collection), (2) methods (gene expression profiling technology), (3) statistical results of differential expression testing (lists of significantly changed genes during onset of labor, direction of change, fold-change value, raw and adjusted *p*-value).

### Data Synthesis

We first retrieved all reported differentially expressed genes based on each study's significance threshold for differential expression with and without term spontaneous labor, and then extracted genes reported in two or more studies within the same tissue. For genes with consistently expressed RNA levels in same direction across different studies of the same tissue, gene ontology (GO) and Kyoto Encyclopedia of Gene and Genomes (KEGG) enrichment analysis, which are effective in clustering the functional genes accordingly to cellular, biological and molecular processes, were conducted using the Database for Annotation, Visualization and Integrated Discovery (DAVID) and g:Profiler (Raudvere et al., 2019). WebGIVI (Sun et al., 2017), a web-based visualization tool, was used to present the network between differentially expressed genes and related pathways. False discovery rate (FDR) was applied to adjust the raw *p*-value for multiple comparisons. Only results with FDR < 0.05 was considered as statistically significant.

## Results

### Study Selection and Basic Characteristics

**Figure 1:**
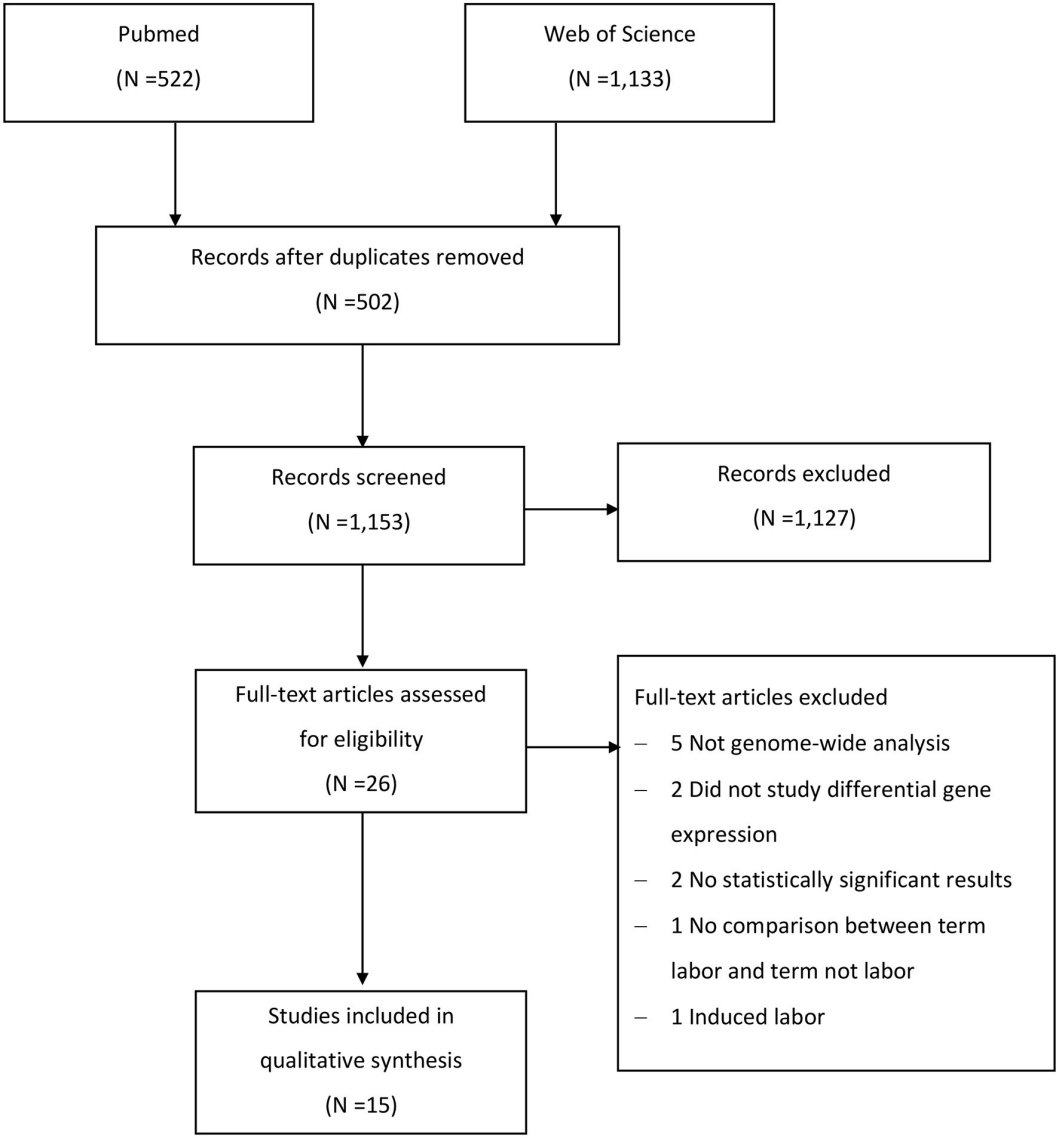
Flow chart of study selection.

We identified 1,655 studies in the two databases, however only 26 publications remained after selection by criteria listed in section Study Selection (Figure 1). Eleven studies were further excluded due to the following reasons: five studies were not genome-wide analysis (Montenegro et al., 2009; Kim et al., 2013; Gomez-Lopez et al., 2017; Lui et al., 2018; Stanfield et al., 2019), differentially expressed genes were not analyzed in two studies (Weiner et al., 2010; Bukowski et al., 2017), two studies had no list of differentially expressed genes due to absence of statistically significant results (Sitras et al., 2008; Montenegro et al., 2009), one study had no comparison between term pregnant women with and those without labor (Makieva et al., 2017), and one study included subjects who required medication to induce labor (Sharp et al., 2016). Eventually, 15 studies (Esplin et al., 2005; Havelock et al., 2005; Bukowski et al., 2006; Haddad et al., 2006; Hassan et al., 2006, 2010; Han et al., 2008; Bollapragada et al., 2009; Lee et al., 2010; Mittal et al., 2010; Peng et al., 2011; Chan et al., 2014; Stephen et al., 2015; Rinaldi et al., 2017; Ackerman et al., 2018), including 266 pregnant women, were included in the final review. A single type of gestational tissue was investigated in 12 of included studies, two types of gestational tissues were investigated in one study (Brown et al., 2004), and three types of tissues were studied in two studies (Lee et al., 2010; Ackerman et al., 2018). The detailed selection procedure was presented in Figure 1.

The basic characteristics of these included studies were shown in Table 1. These studies were conducted between 2004 and 2017 and sample sizes ranged from 10 to 39. The most studied gestational tissue was myometrium (*n* = 7), followed by the uterine cervix (*n* = 4), amnion (*n* = 2), decidua (*n* = 2), placenta (*n* = 2), maternal blood (*n* = 1), fetal blood (*n* = 1), and uterine fundus (*n* = 1). Not surprisingly, the methods of procuring the same type of tissue did vary, *albeit* slightly, across the concerned studies. Myometrial samples in the above studies (Esplin et al., 2005; Havelock et al., 2005; Bukowski et al., 2006; Bollapragada et al., 2009; Mittal et al., 2010; Chan et al., 2014; Ackerman et al., 2018) were obtained from the upper lip of the uterine incision during the surgery or after delivery. Cervical samples were obtained from the anterior lip of the cervix through vagina after delivery (Bukowski et al., 2006; Hassan et al., 2006, 2010; Bollapragada et al., 2009). Amniotic samples in one study were obtained from at least 2 cm apart from the rupture site of the fetal membrane and the margin of the placenta by blunt dissection (Han et al., 2008). Decidual samples were isolated from the fetal membrane by manual separation (Stephen et al., 2015; Rinaldi et al., 2017). Placental samples were obtained from the central area (Lee et al., 2010; Peng et al., 2011). Maternal blood (5 mL) was collected by venipuncture following the delivery of placenta (Peng et al., 2011), fetal blood was collected immediately after the umbilical cord was clamped and cut (Peng et al., 2011), and uterine samples were obtained from the outside surface of the uterine fundus (Bukowski et al., 2006). There was no detailed information on the sample collection in the remaining studies.

**Table 1:** Basic characteristics of included studies.

**Authors**	**Groups**	**No. of subjects**	**Gestational weeks at delivery**	**Mode of delivery**	**Sample types**	**Sample location**	**No. of differentially expressed genes**			**Profiling methods**	**Profiling platform**	**No. of genes measured/ sequencing depth**	**Definition of DEGs**	**Validation methods**
							**Upregulated**	**Downregulated**	**Total**					
Ackerman et al. (2018)	Labor	5	39 (38–41)^a^	CS	Myometrium	Upper lip of incision	16	23	39	RNA-seq#	HiSeq 2500	300 million to 4 billion reads per run	FDR < 0.1 & FC ≥ 1.5	qPCR
	No labor	5	39 (39–39)^a^	CS										
Bollapragada et al. (2009)	Labor	9	40.3 (39.1–41.1)^a^	CS	Myometrium	Upper lip of incision	110	29	139	Microarray	HG-U133 plus 2	Targeting 19, 886 genes	FDR < 1 & FC ≥ 1.5	qPCR
	No labor	9	39 (38.6–39.4)^a^	CS	Cervix	Anterior lip of cervix								
Bukowski et al. (2006)	Labor	7	39.1 ± 0.5^c^	CS	Myometrium	Upper lip of incision	138	362	500	Microarray	HG-U95A	Over 60,000 genes	500 genes with lowest *p*-values	qPCR
	No labor	6	39.1 ± 0.5^c^	CS	Cervix	Anterior lip of cervix	104	396	500					
					Uterine fundus	Outside surface	143	357	500					
Chan et al. (2014)	Labor	5	38–40^d^	SVD	Myometrium	Knife biopsy from the incision	427	337	764	RNA-seq	Genome Analyzer IIx	Over 640 million reads per run	FDR < 0.01	NA
	No labor	5	38–40^d^	CS										
Esplin et al. (2005)	Labor	5	>37	CS	Myometrium	Upper lip of incision	22	34	56	Microarray	Unspecified	6,912 clones in duplicate	FDR < 0.05	qPCR & Northern blot
	No labor	5	>37	CS										
Havelock et al. (2005)	Labor	4	38–42^d^	CS	Myometrium	NA	42	0	42	Microarray	UniGEM V version 2.0	9,182 cDNA	FC ≥ 3	qPCR
	No labor	4	35–39.5^d^	CS										
Mittal et al. (2010)	Labor	19	39.3 (37–41.3)^b^	CS	Myometrium	Upper lip of incision	-	-	471	Microarray	HumanHT-12 v3	Over 48,000 probes	FDR < 0.05 & FC > 1.5	qPCR & ELISA
	No labor	20	38.7 (37–41.9)^b^	CS										
Hassan et al. (2006)	Labor	9	39 (37–41)^b^	SVD	Cervix	Anterior lip of cervix	46	4	50	Microarray	HG-U133 plus 2	Targeting 19,886 genes	FDR < 0.05 & FC > 2	qPCR
	No labor	7	39 (37–39)^b^	CS										
Hassan et al. (2010)	Labor	8	39 (37–40)^b^	SVD	Cervix	Anterior lip of cervix	3	0	3	Microarray#	miRCURY LNA array	455 miRNAs	FDR < 0.05	qPCR
	No labor	9	39 (38–40)^b^	CS										
Rinaldi et al. (2017)	Labor	8	40.1 (38.2–41.1)^b^	SVD/CS	Decudua	Isolated from fetal membranes	56	48	104	Microarray	Human HT-12 v4	Over 48,000 probes	*P* < 0.01 & FC ≥ 1.2	qPCR
	No labor	11	39.3 (39.0–41.0)^b^	CS										
Stephen et al. (2015)	Labor	11	40 (38–42)^b^	SVD	Decidua	Isolated from fetal membranes	396	400	796	Microarray	HG-U133 plus 2	Targeting 19,886 genes	(PPLR ≥ 0.997 OR PPLR ≤ 1 × 10^−5^) & FC ≥ 1.3	qPCR
	No labor	12	39 (37–41)^b^	CS										
Han et al. (2008)	Labor	10	NA	NA	Amnion	2 cm from rupture site and placental margin	15	2	17	Microarray	HG-U133 plus 2	Targeting 19,886 genes	Adjusted *p* < 0.05 & FC > 2	qPCR
	No labor	10	NA	NA										
Haddad et al. (2006)	Labor	12	40.2 (39.2–40.7)^a^	SVD	Amnion	NA	116	81	197	Microarray	HG-U133A	Over 60,000 gene	*P* ≤ 0.02 & FC ≥ 1.4	qPCR
	No labor	12	39.0 (38.7–39.2)^a^	CS							HG-U133B	Over 39,000 genes		
Peng et al. (2011)	Labor	9	38.8 ± 1.0^c^	SVD	Placenta	Central maternal side	20^*^	-	20	Microarray	GMRCL Human 7K set, Version 2	Over 7,000 genes	*P* < 0.05	qPCR
	No labor	9	38.4 ± 0.5^c^	CS	Maternal blood	-	20^*^	-	20					
					Cord blood	-	20^*^	-	20					
Lee et al. (2010)	Labor	7	40.1 ± 1.0^c^	SVD	Placenta	Central area	344	7	351	Microarray	Codelink Bioarray	About 55,000 genes	FDR < 0.05 & FC ≥ 2	qPCR
	No labor	16	39.0 ± 0.9^c^	CS										

*CS, cesarean section; SVD, spontaneous vaginal delivery; FDR, false discovery rate; FC, fold change; PPLR, probability of positive log ratio*.

*RNA-seq, RNA sequencing*;

# *investigated miRNAs*;

* *only listed the top 20 upregulated genes; NA, not available*.

a *Data presented as median (interdquartile range)*.

b *Data presented as median (range)*.

c *Data presented as Mean ± SD*.

d *Data presented as range*.

Microarray was used in 13 of the 15 included studies, and RNA-seq was used in the other two studies. The significance threshold for differential expression was different in each study, and most of them were based on the raw or adjusted *p*-value and fold-change value (Table 1). Non-protein coding miRNAs but not mRNA were investigated in two studies (Hassan et al., 2010; Ackerman et al., 2018), so they were not included in the subsequent functional analysis based on protein-coding mRNA.

### Synthesis of Results

**Figure 2:**
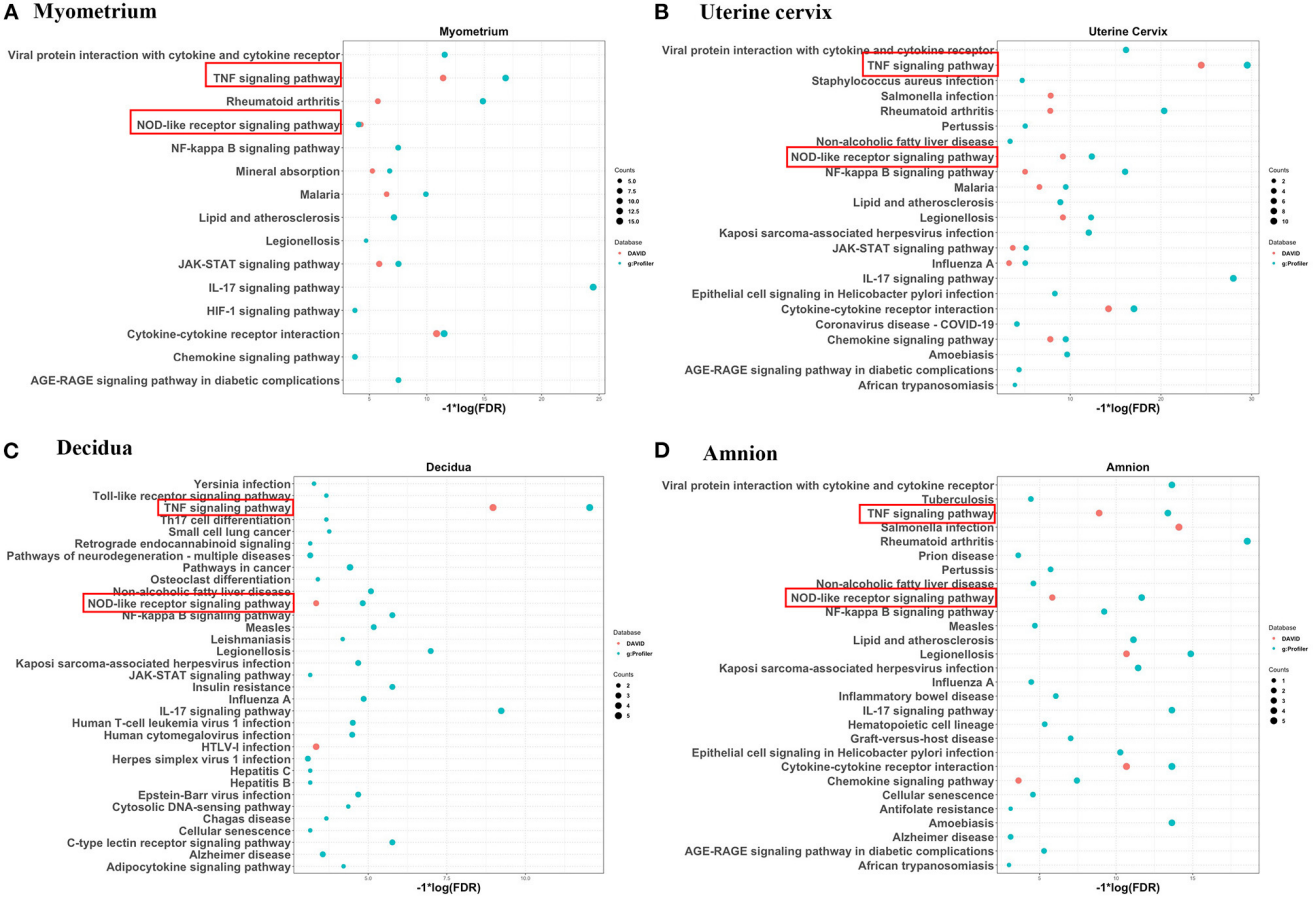
Significantly overrepresented KEGG pathways for significantly increased genes identified by two or more studies in myometrium **(A)**, uterine cervix **(B)**, decidua **(C)**, and amnion **(D)** using DAVID and g:Profiler. KEGG pathways overrepresented in four mentioned gestational tissues were marked by red rectangle. Red dots represent enriched pathways identified by DAVID and blue dots represent pathways identified by g:Profiler. Size of dots represents the gene counts in our gene list involved in the corresponding pathways.

**Figure 3:**
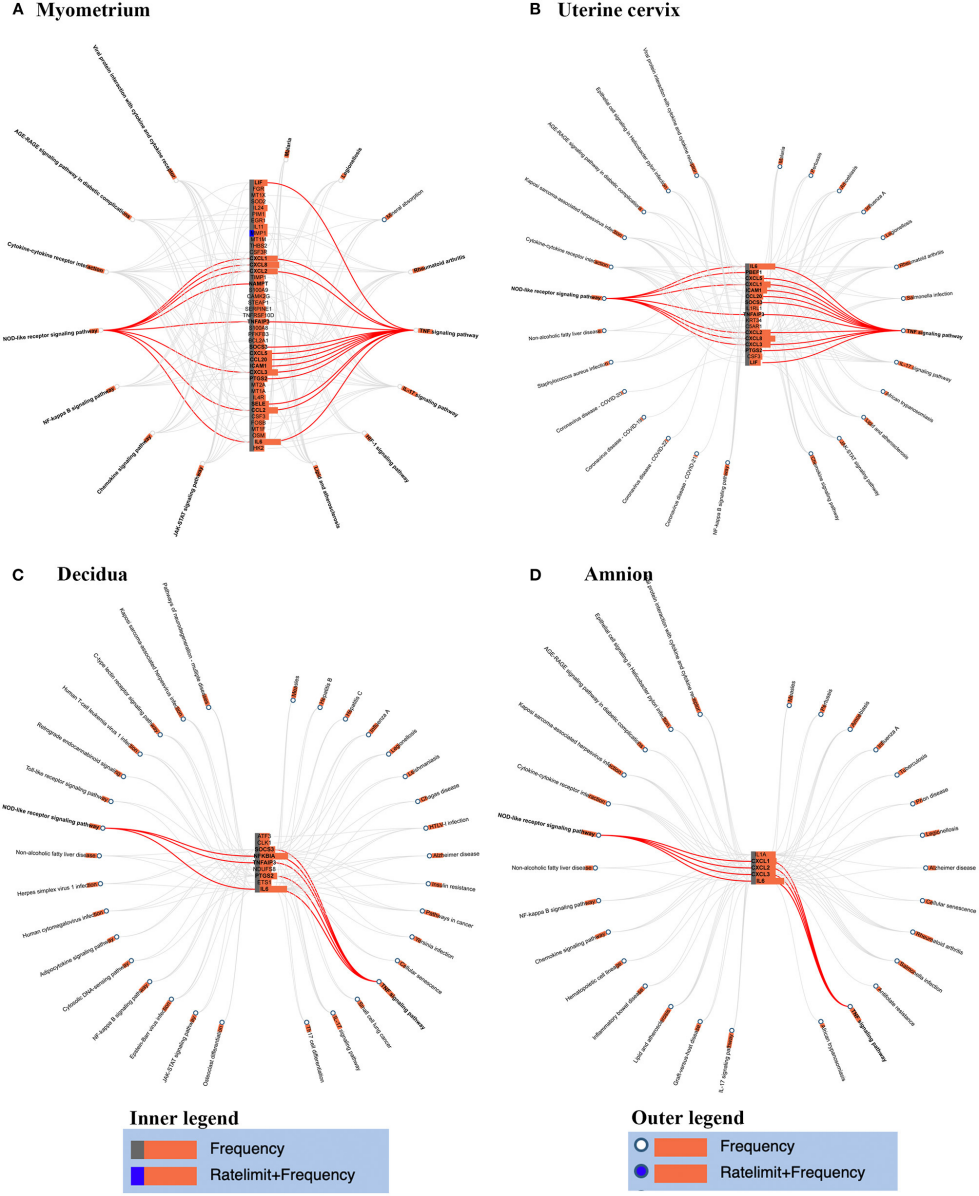
Genes-pathways network in myometrium **(A)**, uterine cervix **(B)**, decidua **(C)**, and amnion **(D)**. Genes involved in the two pathways (i.e., TNF signaling pathway and NOD-like receptor signaling pathway) which enriched in the four tissues were highlighted by red line.

**Figure 4:**
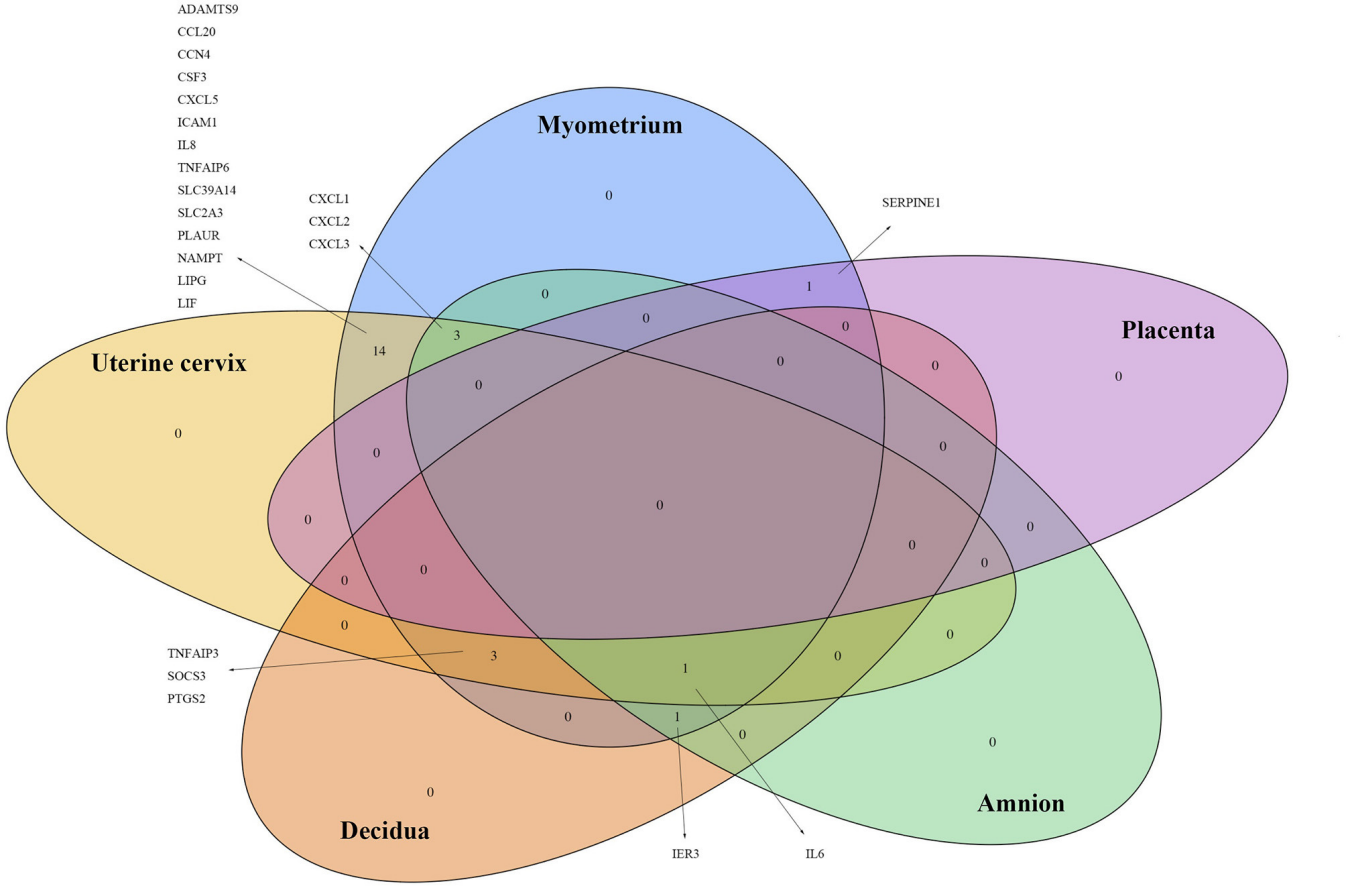
Genes of which the expressions were reported significantly changed in more than one tissue by two or more studies.

**Figure 5:**
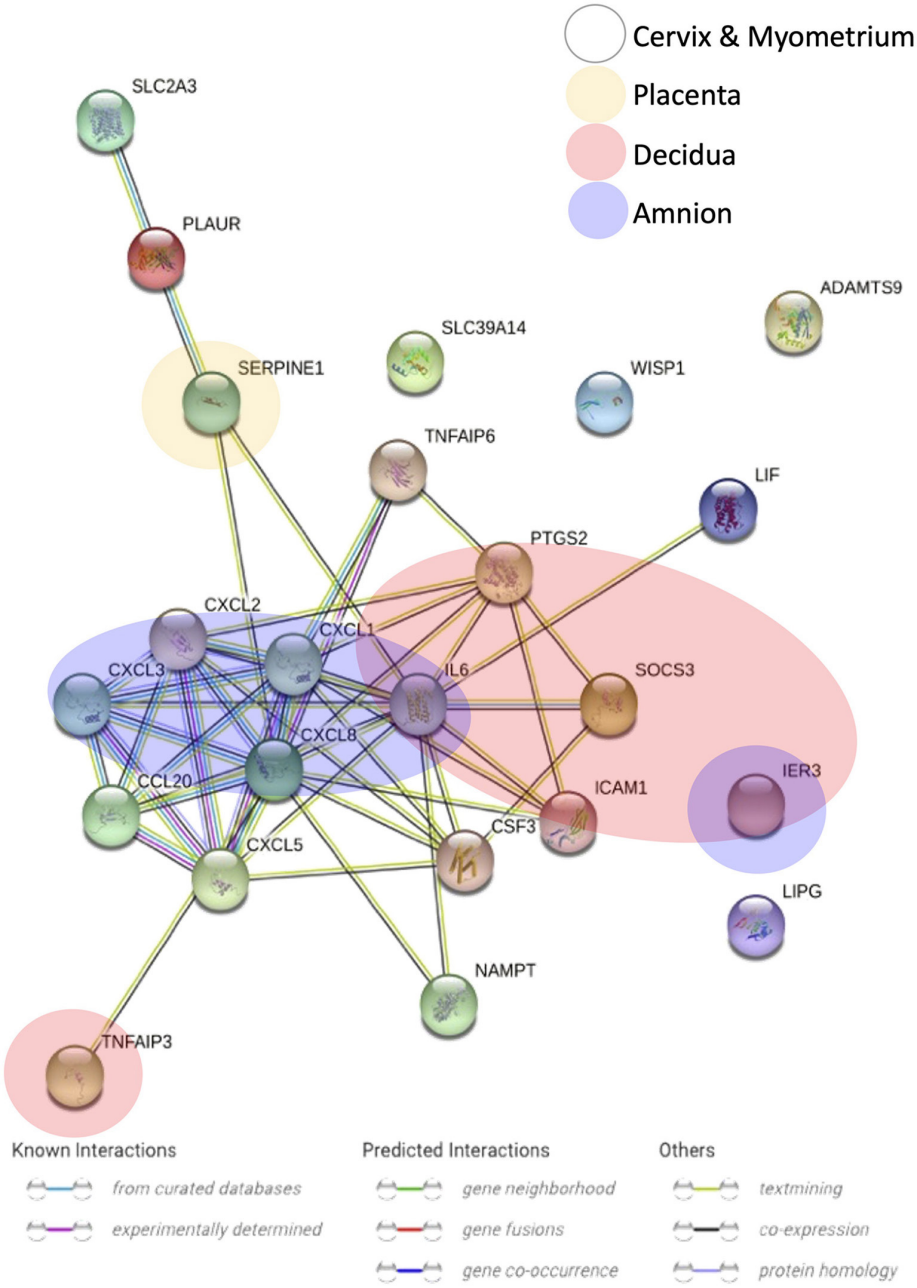
Gene-gene interaction analysis of the differentially expressed genes that overlapped across gestational tissues. Nodes represent genes, edges represent gene-gene association, and colors of edges stand for association evidence in the STRING database.

A total of 3,187 unique differentially expressed genes were reported in the 13 studies. Of these, only 421 genes (13.2%) were reported in two or more studies. For each gene in each type of gestational tissue, its reported direction of changes in RNA levels during labor across different studies was compared (Tables 2–6). Only genes with consistently increased or decreased RNA levels were grouped into five final gene lists (labor-associated gene lists), one for each type of tissue, for statistical comparison with the entire list of genes in the human genome (reference gene list). For each gestational tissue, gene annotation terms (e.g., GO terms such as biological process; Supplementary Material) or gene-associated pathways (e.g., KEGG pathways; Figure 2) that were overrepresented in a labor-associated gene list compared with the reference gene list were identified. Results across five types of gestational tissues were summarized (Figures 2–5).

**Table 2:** Genes with RNA levels reported by two or more studies to be significantly changed in the myometrium during labor.

**Gene**	**No. of studies**	Ackerman et al. (2018)^*^	Bollapragada et al. (2009)^*^	Bukowski et al. (2006)^*^	Chan et al. (2014)^*^	Esplin et al. (2005)^*^	Havelock et al. (2005)^*^	Mittal et al. (2010)^*^
**Genes with consistently increased expression across studies**								
*PTGS2*	4	-	20.25	-	8.98	-	4.00	3.86
*AEBP1*	3	-	-	2.01	1.97	-	-	1.98
*ANGPTL4*	3	-	3.93	-	29.83	-	-	3.04
*AQP9*	3	-	8.79	-	16.92	-	-	3.55
*CEBPD*	3	-	5.66	1.36	4.71	-	-	-
*FOSB*	3	-	5.48	-	26.66	-	3.80	-
*LIF*	3	-	11.89	1.27	15.84	-	-	-
*LILRA5*	3	-	6.01	-	17.81	-	-	3.48
*PIM1*	3	-	-	1.48	4.32	-	-	2.35
*S100A9*	3	-	-	-	13.07	-	3.00	3.52
*SELE*	3	-	13.02	-	54.48	-	5.70	-
*SERPINE1*	3	-	-	1.49	2.54	5.60	-	-
*SERPINE2*	3	-	-	1.42	4.98	2.30	-	-
*SLC16A10*	3	-	8.75	-	12.83	-	-	2.45
*SLC16A3*	3	-	3.07	1.82	4.80	-	-	-
*TRIB1*	3	-	3.35	-	3.30	-	-	2.56
*ACSL4*	2	-	3.02	-	2.31	-	-	-
*ADAMTS9*	2	-	5.30	-	4.21	-	-	-
*ALPL*	2	-	3.42	-	4.44	-	-	-
*B4GALT3*	2	-	-	-	1.79	-	-	1.59
*BCL2A1*	2	-	22.26	-	18.42	-	-	-
*BDNF*	2	-	-	2.21	-	1.70	-	-
*CCL2*	2	-	6.94	-	11.39	-	-	-
*CCL20*	2	-	20.21	-	11.25	-	-	-
*CCN4*	2	-	6.03	-	5.10	-	-	-
*CHI3L1*	2	-	29.95	-	56.19	-	-	-
*CSF3*	2	-	13.83	-	169.48	-	-	-
*CSF3R*	2	-	5.28	-	9.04	-	-	-
*CXCL1*	2	-	17.54	-	20.16	-	-	-
*CXCL2*	2	-	15.77	-	21.28	-	-	-
*CXCL3*	2	-	21.71	-	155.25	-	-	-
*CXCL5*	2	-	33.74	-	584.10	-	-	-
*CXCL8*	2	-	47.91	-	-	-	-	10.36
*DIO3*	2	-	5.61	-	-	-	-	2.52
*DUSP5*	2	-	-	-	-	1.90	-	2.13
*EGR1*	2	-	4.10	-	4.26	-	-	-
*ENC1*	2	-	-	-	2.76	-	-	1.70
*FBN2*	2	-	-	1.87	-	1.20	-	-
*FCAR*	2	-	5.46	-	15.40	-	-	-
*FGF7*	2	-	-	1.98	4.09	-	-	-
*FGR*	2	-	4.47	-	6.72	-	-	-
*FPR1*	2	-	5.80	-	-	-	-	3.02
*GK*	2	-	5.83	-	6.19	-	-	-
*GREM1*	2	-	-	-	9.86	-	-	3.22
*HK2*	2	-	-	-	8.55	-	-	1.64
*ICAM1*	2	-	5.95	-	5.16	-	-	-
*IER3*	2	-	-	-	4.73	-	4.30	-
*IL11*	2	-	12.11	-	31.26	-	-	-
*IL24*	2	-	11.25	-	39.89	-	-	-
*IL4R*	2	-	-	-	3.39	-	-	1.83
*IL6*	2	-	14.11	-	24.56	-	-	-
*ITPRIP*	2	-	-	-	3.08	-	-	2.15
*LIMK2*	2	-	-	2.43	2.12	-	-	-
*LIPG*	2	-	4.82	-	20.49	-	-	-
*LMCD1*	2	-	-	-	3.81	2.70	-	-
*MCEMP1*	2	-	-	-	66.53	-	-	4.10
*MIDN*	2	-	-	-	2.39	-	-	1.70
*MMP1*	2	-	39.08	-	63.96	-	-	-
*MT1A*	2	-	-	-	88.49	-	-	3.56
*MT1F*	2	-	7.93	-	-	-	3.00	-
*MT1M*	2	-	11.72	-	14.14	-	-	-
*MT1X*	2	-	-	-	14.03	-	-	2.82
*MT2A*	2	-	-	-	27.00	-	-	3.41
*MT3*	2	-	-	2.05	7.32	-	-	-
*MXD1*	2	-	-	-	2.90	-	-	1.99
*NAMPT*	2	-	-	-	10.12	-	-	2.68
*NFE2*	2	-	5.77	-	16.97	-	-	-
*NNMT*	2	-	-	-	3.92	-	-	1.87
*NOCT*	2	-	-	-	2.79	-	-	1.90
*NR4A1*	2	-	-	-	4.99	-	4.90	-
*OSM*	2	-	6.30	-	30.25	-	-	-
*PDE4B*	2	-	4.29	-	-	-	-	2.27
*PDPN*	2	-	-	-	3.26	-	-	2.09
*PFKFB3*	2	-	-	-	4.10	-	-	1.60
*PLA2G2A*	2	-	-	1.83	10.33	-	-	-
*PLAUR*	2	-	5.77	-	5.88	-	-	-
*PLK3*	2	-	-	1.87	2.98	-	-	-
*PROK2*	2	-	21.01	-	16.38	-	-	-
*PTGES*	2	-	4.42	-	12.09	-	-	-
*PTX3*	2	-	13.64	-	19.85	-	-	-
*RARRES1*	2	-	10.41	-	10.94	-	-	-
*RUNX1*	2	-	-	-	5.57	1.50	-	-
*S100A8*	2	-	-	-	19.03	-	-	3.85
*SAA1*	2	-	-	-	31.67	-	12.00	-
*SEMA6B*	2	-	2.85	-	3.40	-	-	-
*SERTAD1*	2	-	-	-	2.40	-	-	1.62
*SLA*	2	-	3.53	1.89	-	-	-	-
*SLC11A1*	2	-	5.53	-	26.98	-	-	-
*SLC2A3*	2	-	3.59	-	3.13	-	-	-
*SLC39A14*	2	-	3.52	-	4.24	-	-	-
*SLC7A5*	2	-	-	-	5.45	-	-	2.74
*SOCS3*	2	-	13.11	-	16.86	-	-	-
*SOD2*	2	-	7.81	-	5.77	-	-	-
*SRPRB*	2	-	-	-	1.77	-	-	1.52
*STEAP1*	2	-	9.38	-	7.67	-	-	-
*TEAD4*	2	-	2.92	-	2.91	-	-	-
*TFPI2*	2	-	9.79	-	9.52	-	-	-
*THBS2*	2	-	-	-	2.69	2.10	-	-
*TIMP1*	2	-	-	-	5.00	1.70	-	-
*TNFAIP3*	2	-	4.04	-	3.18	-	-	-
*TNFAIP6*	2	-	11.37	-	7.38	-	-	-
*TNFRSF10D*	2	-	3.12	-	3.71	-	-	-
**Genes with consistently decreased expression across studies**								
*ANGPTL1*	3	-	−3.46	-	−2.71	-	-	−1.59
*CYP4B1*	3	-	−5.03	-	−10.18	-	-	−2.73
*FAXDC2*	3	-	−4.18	-	−4.41	-	-	−2.28
*PLCL1*	3	-	−6.98	-	−8.63	-	-	−2.57
*SELENOP*	3	-	-	-	−4.38	−2.10	-	−2.46
*ABLIM1*	2	-	-	-	−2.02	−1.80	-	-
*ALDH1A2*	2	-	-	−1.62	−4.20	-	-	-
*ARHGEF6*	2	-	-	-	−2.61	-	-	−1.60
*BIN1*	2	-	-	−1.56	-	−2.00	-	-
*CACNB2*	2	-	-	−1.77	−2.24	-	-	-
*CAMK2G*	2	-	-	−1.50	−2.43	-	-	-
*DAAM1*	2	-	-	−1.57	−2.41	-	-	-
*EMX2*	2	-	-	-	−2.17	-	-	−1.58
*EPM2A*	2	-	-	−1.95	−2.10	-	-	-
*GCOM1*	2	-	−3.45	-	-	-	-	−1.72
*GPR161*	2	-	-	−1.83	−4.34	-	-	-
*GPR37*	2	-	-	-	−13.14	-	-	−2.20
*HADH*	2	-	-	-	−3.06	-	-	−2.13
*HOXA11*	2	-	-	-	−2.21	-	-	−1.81
*KCNH2*	2	-	-	-	-	−1.50	-	−2.29
*LGMN*	2	-	-	-	-	−1.70	-	−1.64
*MAMDC2*	2	-	−4.65	-	−7.27	-	-	-
*MITF*	2	-	-	-	−3.28	-	-	−1.54
*OMD*	2	-	−2.54	-	−5.05	-	-	-
*PCDH18*	2	-	-	-	−3.40	-	-	−1.78
*PCYOX1*	2	-	−2.60	-	−3.21	-	-	-
*PDZRN4*	2	-	−4.57	-	−6.48	-	-	-
*PHACTR2*	2	-	−6.22	-	−2.34	-	-	-
*PPM1A*	2	-	-	−2.09	−1.85	-	-	-
*PPP1R3C*	2	-	−3.03	-	−2.61	-	-	-
*PTGER3*	2	-	-	-	−7.09	-	-	−2.24
*RAI2*	2	-	-	-	−2.80	-	-	−1.55
*RERG*	2	-	-	-	−3.73	-	-	−2.52
*ZNF189*	2	-	-	-	−2.58	-	-	−1.85
**Genes with conflicting direction of change in expression across studies**								
*HLF*	3	-	−3.05	1.63	−7.53	-	-	-
*KCNK3*	3	-	6.34	−2.57	6.96	-	-	-
*DAPP1*	2	-	-	-	−3.85	-	3.60	-
*HSPA6*	2	-	-	−1.81	6.56	-	-	-
*IFI6*	2	-	-	−1.22	-	-	6.20	-
*IFIT1*	2	-	−2.91	-	-	-	6.90	-
*MMP17*	2	-	-	−1.61	3.13	-	-	-
*WT1*	2	-	-	2.41	-	−1.40	-	-

* *Show in column 3 onwards are fold-change value of RNA levels during labor. A positive value represents an increased RNA level during labor, while a negative value represents a decreased level*.

**Table 3:** Genes of which RNA levels were reported by two or more studies to be significantly increased in uterine cervix during labor.

**Gene**	**No. of studies**	Bollapragada et al. (2009)^*^	Bukowski et al. (2006)^*^	Hassan et al. (2006)^*^	Hassan et al. (2010)^*^
*ADAMTS9*	2	5.64	-	5.75	-
*C5AR1*	2	6.15	-	5.52	-
*CCL20*	2	19.91	-	15.45	-
*CCN4*	2	22.90	-	9.05	-
*CSF3*	2	27.89	-	7.19	-
*CXCL1*	2	5.03	-	21.72	-
*CXCL2*	2	19.45	-	25.65	-
*CXCL3*	2	36.45	-	11.05	-
*CXCL5*	2	11.09	-	28.24	-
*CXCL8*	2	11.90	-	26.64	-
*ICAM1*	2	8.10	-	5.39	-
*IL1RL1*	2	10.97	-	9.82	-
*IL6*	2	49.50	-	22.36	-
*KRT34*	2	10.70	-	13.09	-
*LIF*	2	37.14	-	11.71	-
*LIPG*	2	6.20	-	7.18	-
*NR4A2*	2	8.43	-	11.62	-
*PBEF1*	2	7.19	-	6.67	-
*PLAUR*	2	6.91	-	7.18	-
*PTGS2*	2	7.10	-	50.48	-
*SLC2A3*	2	13.87	-	11.30	-
*SLC39A14*	2	10.42	-	7.26	-
*SOCS3*	2	12.82	-	11.66	-
*STC1*	2	17.54	-	11.50	-
*TNFAIP3*	2	8.29	-	5.92	-
*TNFAIP6*	2	18.62	-	14.30	-

* *Show in column 3 onwards are fold-change value of RNA levels during labor. A positive value represents an increased RNA level during labor, while a negative value represents a decreased level*.

**Table 4:** Genes of which RNA levels were reported by two or more studies to be significantly increased or decreased in decidua during labor.

**Gene**	**No. of study**	Rinaldi et al. (2017)^*^	Stephen et al. (2015)^*^
*ATF3*	2	2.31	1.55
*CLK1*	2	1.55	2.03
*ETS1*	2	1.45	2.25
*GEM*	2	1.72	3.42
*HNRNPA2B1*	2	1.43	1.95
*IER3*	2	2.68	2.97
*IL6*	2	2.66	19.56
*NDUFS8*	2	−1.21	−2.32
*NFKBIA*	2	1.99	2.05
*PELI1*	2	1.36	4.82
*PHLDA1*	2	2.15	6.81
*PTGS2*	2	2.47	3.01
*SLC20A1*	2	1.43	4.62
*SOCS3*	2	1.33	1.76
*TNFAIP3*	2	2.72	3.52

* *Show in column 3 onwards are fold-change value of RNA levels during labor. A positive value represents an increased RNA level during labor, while a negative value represents a decreased level*.

**Table 5:** Genes of which RNA levels were reported by two or more studies to be significantly increased in amnion during labor.

**Gene**	**No. of study**	Haddad et al. (2006)^*^	Han et al. (2008)^*^
*CXCL1*	2	5.70	10.80
*CXCL2*	2	6.50	88.50
*CXCL3*	2	6.50	163.80
*HAS1*	2	1.60	31.10
*IER3*	2	4.30	7.30
*IL1A*	2	2.10	12.40
*IL6*	2	5.30	36.10

* *Show in column 3 onwards are fold-change value of RNA levels during labor. A positive value represents an increased RNA level during labor, while a negative value represents a decreased level*.

**Table 6:** Genes of which RNA levels were reported by two or more studies to be significantly increased in placenta during labor.

**Gene**	**No. of study**	Lee et al. (2010)^*^	Peng et al. (2011)^*^
*ADM*	2	3.57	1.49
*DAG1*	2	2.76	1.31
*SERPINE1*	2	2.25	1.54

* *Show in column 3 onwards are fold-change value of RNA levels during labor. A positive value represents an increased RNA level during labor, while a negative value represents a decreased level*.

### Myometrium

Myometrium was investigated in 6 mRNA studies. The expression of 144 genes were reported to be changed in women with labor compared to those without labor in two or more studies (Table 2). Of these, 102 genes showed consistently increased (Table 2, first section) and 34 genes showed decreased (Table 2, second section) mRNA expression across multiple studies. However, the remaining eight genes showed inconsistent direction of change in RNA levels during labor across studies (Table 2, third section), which were not further analyzed.

Of the genes with consistently increased RNA levels in the myometrium during labor, *PTGS2* was reported in four of the six relevant studies (66%), whereas *AEBP1, ANGPTL4, AQP9, CEBPD, FOSB, LIF, LILRA4, PIM1, S100A9, SELE, SERPINE1, SERPINE2, SLC16A10, SLC16A3*, and *TRIB1* were reported in three studies (50% of the relevant studies) (Table 2, first section). Of the genes which were observed to be expressed at decreased RNA levels in the myometrium during labor, *ANGPTL1, CYP4B1, FAXDC2, PLCL1*, and *SELENOP* were reported in three studies (50% of the relevant studies), whereas the remaining 29 genes were reported in two studies (Table 2, second section). DAVID and g:Profiler, web server for functional enrichment analysis, were used to gain insight into the biology of these consistently changed genes. DAVID showed that 25 biological processes, such as inflammatory response, neutrophil chemotaxis and response to interleukin 1 (Supplementary Material 1), and seven pathways, including TNF signaling pathway, NOD-like receptor signaling pathway, Jak-STAT signaling pathway and cytokine-cytokine receptor interaction pathway (Figure 2A, red dots) were significantly overrepresented in this comparison. While analysis in g:Profiler demonstrated 390 biological processes (Supplementary Material 2) and 15 pathways (Figure 2A, blue dots) were enriched in the same comparison. Among the 10 biological processes with the lowest FDR, eight biological processes were related to the immune/inflammatory response, and the other two biological processes were associated with zinc ion. Enriched pathways identified by DAVID were also identified by g:Profiler (Figure 2A).

### Cervix

There were 26 genes reported in two or more studies (maximum three studies) to show changed RNA levels in the cervix during labor. Across the multiple studies, the RNA levels of all 26 genes were consistently higher in women with labor than those without (Table 3). Compared with the entire list of genes in the human genome, in DAVID, this 26-gene list was overrepresented with 21 biological processes, including inflammatory response, immune response and chemokine-mediated signaling pathway (Supplementary Material 3), and overrepresented with 11 pathways, including those associated with TNF, NOD-like receptor and chemokine signaling (Figure 2B, red dots). While in g:Profiler, this list was enriched in 266 biological processes, among which 10 biological processes with the lowest FDR were parts of immune/inflammatory response (Supplementary Material 4) and 22 pathways, of which 10 pathways were shared with the analysis using DAVID (Figure 2B, blue dots).

### Decidua

Gene expression profile in decidua was investigated in two included studies. Fifteen genes were identified to be differentially and consistently changed during labor by both studies. All the direction of changes were upregulated, except that of *NDUFS8* which was downregulated (Table 4). DAVID analysis showed three overrepresented pathways (Figure 2C, red dots) but no enriched biological processes; while analysis using g:Profiler showed the 15-gene list was enriched with 263 biological processes (Supplementary Material 5) and 32 pathways (Figure 2C, blue dots) in decidua. TNF and NOD-like receptor signaling pathway were identified by two databases.

### Amnion

Of the two studies on amnion, mRNA levels of seven genes were reported to be significantly and consistently increased by both included studies. Using DAVID, GO analysis showed five overrepresented biological processes which belong to the immune/inflammatory response (Supplementary Material 6) and pathway analysis revealed six overrepresented pathways (Figure 2D, red dots). Enrichment analysis in g:Profiler showed the 7-gene list in amnion was enriched in 224 biological processes, including immune cell migration and response to cytokine/chemokine, (Supplementary Material 7) and 27 pathways (Figure 2D, blue dots). Five pathways, TNF, NOD-like receptor, legionellosis, cytokine-cytokine receptor interaction, and chemokine signaling pathways, were identified by both DAVID and g:Profiler (Figure 2).

### Placenta

Besides, there were three genes identified to be differentially expressed in the two studies on placenta, and mRNA levels of them were consistently increased during labor (Table 6). No significantly overrepresented biological processes or pathways related to the three genes in placenta were found in DAVID. While 189 biological processes, including cytokine production and regulation of cytokine production, were enriched (Supplementary Material 8), but no pathways were enriched, in g:Profiler.

As gene expression in uterine fundus, maternal blood and cord blood was investigated in only one single study, we could not make further comparison for these tissues.

### Comparison of Results From Myometrium, Cervix, Amnion, Decidua, and Placenta

We also searched for any overlapping of the reported differentially expressed genes across reviewed gestational tissues. A total of 23 genes were identified to be differentially expressed, between women with labor and without labor, in more than one tissue and by two or more studies (Figure 4). All the expressions were reported to be upregulated. No genes were shared across all the five reviewed tissues. *IL6* was the only gene expression that was significantly changed in all tissues, except placenta. Seven genes, *CXCL1, CXCL2, CXCL3, TNFAIP3, SOCS3, PTGS2*, and *IER3*, were reported in three of the five reviewed tissues. The remaining 14 genes were reported in two of the reviewed tissues. To visualize how these overlapping gene were related, Search Tool for Retrieval of Interacting Genes/Proteins (STRING) with a high-confidence score (interaction score ≥ 0.90) comparing with a genome background and the evidence mode in the settings were performed (Szklarczyk et al., 2019). The potential interactions are shown in Figure 5. Nodes represent genes, edges stand for the gene-gene associations and the line color indicates the type of interaction evidence in the STRING database. Notably, *IL6* was the central node in the network.

### Functional Enrichment Analysis

To gain insight into the spatial differences of biology, we summarized the similarities and differences of the overrepresented pathways across the reviewed gestational tissues. No enriched pathways related to placenta were identified by either database. TNF and NOD-like receptor signaling pathways were overrepresented in all reviewed tissues except placenta by both databases (red rectangles in Figure 2). To better visualize the association between the identified genes and related pathways in each tissue, the gene-pathway network was created (Figure 3), showing that *IL6* was the only gene reported in the four mentioned tissues and related to these two pathways (Figure 3). Mineral absorption pathway, involving *MT1A, MT1M, MT1F, MT2A*, and S*TEAP1*, was identified by two databases but was overrepresented in only myometrium (Figures 2, 3).

## Discussion

Initiation of human parturition entails a complex, multifactorial set of events, and the mechanism of which remains barely understood. To provide an overview of the current literature on human labor transcriptomics, we conducted a systematic review analyzing genome-wide studies of gestational tissues in healthy pregnancies.

### Principal Findings

Principal findings of this systematic review were: (1) *IL6*, the most reported differentially expressed gene during labor in our included studies, was identified to be significantly upregulated in seven studies across four gestational tissues and was the central gene among the overlapped genes across gestational tissues; (2) Pathway analysis indicated TNF and NOD-like receptor signaling pathways overrepresented in the myometrium, cervix, decidua and amnion. *IL6* was the only gene reported in the four tissue types and involved in the two pathways. (3) Metal ion-associated annotation terms, such as response to zinc and cadmium ion and mineral absorption pathway, were overrepresented in only myometrium.

### Role of IL6 in the Onset of Human Labor

Interleukin 6 is a well-known marker with upregulated expression in preterm birth, premature rupture of membrane (PROM), and term labor (Febbraio and Pedersen, 2005). In this review, of the 13 studies in which the differential expression of protein-coding mRNA was investigated, seven studies reported the increased mRNA levels of *IL6* across the myometrium, cervix, decidua and amnion (Haddad et al., 2006; Hassan et al., 2006; Han et al., 2008; Bollapragada et al., 2009; Chan et al., 2014; Stephen et al., 2015; Rinaldi et al., 2017). However, no change in mRNA levels was reported in the two studies on placenta (Lee et al., 2010; Peng et al., 2011). Inconsistent findings on the *IL6* expression in laboring placenta have been discussed in previous review (Hadley et al., 2018). Overall, IL6 is a common cytokine in most of gestational tissues with increased expression during labor. IL6 is a pleiotropic cytokine with broad effects on immune system, including promoting proliferation of lymphocytes and regulating acute-phase response (Hunter and Jones, 2015). Interestingly, IL6 has both pro- and anti-inflammatory properties (Scheller et al., 2011).

Figure 5 showed the potential associations among overlapped differentially expressed genes across gestational tissues. *IL6* was the central gene and linked with multiple genes. *IL6* linked with, previously demonstrated to induce, a cluster of chemokines, such as *CXCL1* and *CXCL8*, which chemoattract immune cells, especially neutrophils (Roy et al., 2012; Caiello et al., 2014). *IL6* also linked with *CSF3*, which stimulates the proliferation and maturation of neutrophils. But the definite association between IL6 and CSF3 is required further investigation. Besides, NAMPT, which inhibits neutrophil apoptosis, increases the production of IL6 (Travelli et al., 2018). Therefore, *IL6* was associated with genes increasing the number and activity of neutrophils. Neutrophils might participate in the human labor by releasing enzymes including MMPs which induce cervical ripening, and by secreting cytokines which further induce the infiltration of immune cells. Moreover, *IL6* upregulates expression of *PTGS2* leading to the production of prostaglandins which induce myometrial contractions (Mitchell et al., 1991). Besides, *IL6* was also associated with *TNFAIP6* and *SOCS3*, which negatively regulate inflammatory response. This suggests that the anti-inflammatory property of *IL6* might also involves in the onset of human labor, and increased expressions of anti-inflammatory genes are of importance to maintain the inflammatory homeostasis during physiological term parturition.

Figure 3 showed the association between genes and related pathways. As shown in Figure 3, TNF and NOD-like receptor signaling pathways were enriched in myometrium, cervix, decidua and amnion, and IL6 were identified to changed significantly during labor and contributed most to the two pathways. Previous studies also investigated the role of IL6 in human labor. Rauk et al. showed that IL6 upregulates the expression of oxytocin receptor and the capacity of binding oxytocin in myometrium explants (Rauk et al., 2001), which induces uterine contraction. Mitchell et al. found that IL6 stimulate production of prostaglandins in a concentration-related manner in decidua and amnion cells (Mitchell et al., 1991). Meisser et al. demonstrated that IL6 increase the activity of MMPs, which is essential in cervical ripening (Meisser et al., 1999). Taken together, we speculate that IL6 might play a determinant role in the onset of human labor via multiple connections with genes across gestational tissues. On the other hand, transcriptomic analysis in opossum showed that parturition was characterized by an inflammatory response consistent with human parturition (Hansen et al., 2016; Griffith et al., 2017). However, the IL6 transcripts were elevated and peaked at parturition and dropped afterward in opossum, but stayed high level in humans (Hansen et al., 2017). The shorter pregnancy and labor process in opossum than humans might contribute to the differences, suggesting a unique process in human labor.

Interleukin 6 can be produced in almost all immune cells in response to microbial molecules, but the mechanism by which how it triggers the secretion of IL6 in normal term labor without evidence of infection remains unclear. Phillippe proposed a “cell-free fetal DNA (cffDNA)/telomore hypothesis” that the increase in cffDNA, which is released by placenta during apoptosis at term, stimulates toll-like receptor 9 leading to production of pro-inflammatory cytokines including IL6 (Phillippe, 2015). However, further studies are needed to investigate this theory and the function of IL6 in human labor.

### Inflammatory and Immune Response in Human Labor

Results of this systematic review confirmed that inflammatory and immune response are involved in most gestational tissues in normal human parturition. GO analysis demonstrated that inflammatory response, immune response, chemokine-mediated signaling pathway and response to lipopolysaccharide were overrepresented in the myometrium, cervix and amnion. *CXCL1, CXCL2* and *CXCL3* were involved in the four biological processes and the mRNA levels were upregulated in the three relevant tissues. Apart from the general concept of inflammatory and immune response, chemokine-mediated signaling pathway comprises a series of molecular events initiated by chemokines binding to its receptors, which is essential for the coordination of cell migration. Overrepresented chemokine-mediated signaling pathway, as well as the increased expression of *CXCL1, CXCL2*, and *CXCL3*, may explain the infiltration of immune cells in gestational tissues during labor (Frim et al., 1988; Majzoub and Karalis, 1999).

Response to lipopolysaccharide refers to any processes resulted from lipopolysaccharide stimuli, a component of cell-wall of Gram-negative bacteria, resulting in the production of pro-inflammatory cytokines (O'Neill and Bowie, 2007). Response to lipopolysaccharide also has been implicated in the pathophysiology of preterm labor, PROM and chorioamnionitis (Romero et al., 1987, 1988). The involvement of this biological process in human labor, both preterm and term, may be due to production of pro-inflammatory cytokines via the above-mentioned mechanisms, such as stimulating secretion of prostaglandins which induce uterine contraction, or release of MMPs which facilitate cervical ripening. Further studies, nevertheless, are required to investigate the mechanisms.

Pathway analysis indicated that TNF and NOD-like receptor signaling pathway were overrepresented in all the reviewed tissues except placenta. Tumor necrosis factor (TNF) induces a broad range of molecular events, including induction of apoptosis, initiation of necrosis and activation of inflammation (Chu, 2013). In addition to the property of pro-inflammation, TNF also stimulates the synthesis of prostaglandins (Christiaens et al., 2008). On the other hand, nucleotide-binding oligomerization domain (NOD) like-receptors are a family of pattern recognition receptors, which are responsible for the detection of pathogen-associated molecular patterns, and the generation and regulation of innate immune system (Jakopin, 2014). These overrepresented biological processes and pathways in most gestational tissues indicated the involvement of inflammatory and/or immune response in the onset of normal human labor.

Besides the common biology defined by the differentially expressed genes during labor onset in most gestational tissues, specific molecular functions or pathways in certain tissue are also important to understand the underlying mechanism that triggers the human parturition.

### Metallothioneins in Myometrium During Labor Onset

Genes encoding metallothioneins, including *MT1A, MT1F, MT1M, MT1X, MT2A*, and *MT3*, and related pathways, such as mineral absorption pathway, cellular response to zinc and cadmium ion, were overrepresented in exclusively the myometrium. Metallothioneins are a family of cysteine-rich, metal-binding proteins, capable of protecting cells against metal toxicity, oxidative stress and apoptosis (Klaassen et al., 1999; Shimoda et al., 2003). Considering the observations that increased infiltration and activity of cytotoxic cells, such as NK cells, in the gestational tissues during labor (Abadia-Molina et al., 1996; Wicherek and Galazka, 2007), the increased metallothioneins in myometrium suggests that metalloproteineins participate in the regulation of homeostasis responsible for restricting the overload of immune response. Also, in this review, upregulated expressions of genes related to metallothioneins were only reported in studies on myometrium, which might suggest that metallothioneins were involved in the myometrial contraction. However, the exact function of metallothioneins in the process of labor requires future study.

### Information From Other Differentially Expressed Genes

The mRNA level of prostaglandin-endoperoxide synthase 2 (PTGS2) was identified to be significantly increased in myometrium, cervix and decidua by eight included studies in our review. PTGS2, also known as COX2, is the rate-limiting enzyme in the synthesis of prostaglandin, which is a strong stimulator of uterine activity (O'Brien, 1995). In addition to the upregulated expression during human labor, delayed parturition in mice lacking *PTGS2* demonstrated the essential role of PTGS2 in initiating the labor (Gross et al., 1998; Loftin et al., 2001).

Adrenomedullin (ADM) gene levels were found to increase during labor in placenta. The expression of ADM was demonstrated to be predominantly expressed in placenta and fetal membrane but less in uterine muscle (Makino et al., 2001). Di lorio et al. reported that the plasma ADM protein level was significantly higher obtained after the labor onset than the concentration before labor, and the production of ADM by fetal membranes cultured cells was greater from samples collected after vaginal delivery than from Cesarean section (Di Iorio et al., 2001). The increased production of ADM throughout pregnancy was speculated to maintain a quiescent uterus, as ADM is a potent smooth muscle relaxant, and progesterone enhances the ADM production (Norwitz et al., 2007). But unlike the functional withdrawal of progesterone, the ADM concentration increased during labor, which was speculated previously to compensate for the increased synthesis and release of local-acting vasoconstrictor substances (e.g., prostaglandins) by acting to dilate vessels and facilitate blood flow during parturition (Al-Ghafra et al., 2003).

Besides, mRNA levels of genes regulating the activity of plasminogen, such as *SERPINE1* and *PLAUR*, were also reported to be changed during labor. Plasminogen activator family, including plasminogen activator urokinase-type (PLAU) and its receptor (PLAUR), is responsible for the MMPs activation (Ny et al., 2002; Curry Jr and Osteen, 2003). Plasminogen activator dependent activation of MMPs leads to activation of plasminogen activator and other MMPs, which further propagates the proteolytic cascade (Ny et al., 2002; Xu et al., 2010). Thus, the increased PLAUR level in cervix during labor facilitate the cervical remodeling and ripening through the activation of MMPs. On the other hand, plasminogen activator inhibitor-1 (SERPINE1) inhibits the activity and leads to the degradation of plasminogen activator (Olson et al., 2001). Expression of *SERPINE1* was also found to be increased in myometrium and placenta during labor, which might be due to the elevated glucocorticoids (Medcalf et al., 1988). Aside from the ability to inhibits plasminogen activator, SERPINE1 also regulates cell adhesion to extracellular matrix, and SERPINE1 in the maternal-fetal interface disrupts the adhesion of cotyledon to caruncle which results in placental separation and expulsion (Burghardt et al., 2009; McNeel et al., 2013).

### Limitations of Current Study

Firstly, heterogeneities in the study design and methods used among included studies make it challenging to compare the differential gene expression in different studies. All the included studies were cross-sectional studies rather than longitudinal and differ in mode of delivery. Regarding the transcriptome profiling, untargeted and unbiased transcriptome profiling methods were used in all included studies, but the sensitivity of profiling platform varies (Table 1). Qualitatively, however, the aggregated outcomes of these included studies demonstrated the most common changes during labor onset across different gestational tissues. Another problem that cannot be ignored is the different standards concerning data availability and differential gene expression criteria in different studies. For instance, several studies didn't report the full list of differentially expressed genes, but top 20 or 50 most significant genes, which may restrict and underpower the functional analysis. Therefore, we might omit information from genes that are important in labor onset but did not meet the threshold for differential expression or did not present in the original study. Aggregating raw data from each included study and analyzing it by the same statistical method would be helpful to adjust this problem. Secondly, only studies on protein-coding RNA can be included in the functional analysis, which might limit the important non-protein coding genes which regulate protein expression at the post-transcriptional level via interaction with targeted mRNAs. Functional analysis of these targeted mRNAs might be a remedy for this missing information. But in our review, only two studies investigated differential expression of miRNAs in myometrium and cervix, respectively. In addition, transcriptomic changes during preterm labor onset were not included in this review. Inflammatory and immune responses implicated in both preterm and term labor onset, but the reason why the timing of labor onset differs remains unclear. Comparing the similarities and differences in transcriptomic changes between preterm and term labor might provide insight. Lastly, in our study the comparisons were made between two groups of women with and without labor, of which differences identified might be inter-individual variation rather than labor-associated changes. Also, samples were obtained after the delivery of fetus/placenta in the current studies, due to the infeasibility of gestational tissues sampling at the early stage of labor, which might only demonstrate the changes at the later stages of labor instead of changes occurring at around the onset of labor. Considering that maternal blood enriches immune cells and can be serially taken during labor onset, transcriptomic studies using maternal blood from women who are about to go into term labor might provide markers to understand the initiation of human labor and to diagnosis labor-associated disorders.

### Conclusion and Implications

The results of this systematic review suggested that immunological and inflammatory regulation are involved in specific gestational tissues during labor onset. TNF and NOD-like receptor signaling pathways were overrepresented in all mentioned tissues, except the placenta. *IL6* was the only gene involved in both pathways and also reported in the four mentioned tissues. *IL6* was the most common reported genes in included studies, and the central gene in the association network of overlapped genes across reviewed tissues. It demonstrates that *IL6* might be the key gene that triggers specific mechanisms in different gestational tissues except placenta. The increased *IL6*, as well as other pro-inflammatory cytokines and chemokines, might lead to the onset of labor through inducing uterine contraction, wakening fetal membranes and stimulating cervical ripening.

## Data Availability Statement

The original contributions presented in the study are included in the article/Supplementary Material, further inquiries can be directed to the corresponding author.

## Author Contributions

WD performed the database search, extracted data, and wrote the manuscript. SC performed the database search and data extraction as a second reviewer, and revised the manuscript. CW performed the database search and data extraction as a third reviewer, and revised the manuscript. CL revised the database search and manuscript. TL performed the final review of the manuscript. All authors contributed to the article and approved the submitted version.

## Funding

This work was supported by the General Research Fund (Project No.: CUHK 14130816) of the Research Grants Council of the Hong Kong SAR Government, China.

## Conflict of Interest

The authors declare that the research was conducted in the absence of any commercial or financial relationships that could be construed as a potential conflict of interest.

## Publisher's Note

All claims expressed in this article are solely those of the authors and do not necessarily represent those of their affiliated organizations, or those of the publisher, the editors and the reviewers. Any product that may be evaluated in this article, or claim that may be made by its manufacturer, is not guaranteed or endorsed by the publisher.
